# Range size and abundance dynamics of Japanese breeding birds over 40 years suggest a potential crisis in warm areas

**DOI:** 10.1038/s41598-025-01382-8

**Published:** 2025-05-19

**Authors:** Yuichi Yamaura, Kazuhiro Kawamura, Masayuki Senzaki, Munehiro Kitazawa, Isao Nishiumi, Naoki Katayama, Tatsuya Amano, Yasushi Ishigooka, Shigeto Sudo, Takeshi Osawa, Mutsuyuki Ueta

**Affiliations:** 1 Shikoku Research Center, Forestry and Forest Products Research Institute, Kochi, Kochi Japan; 2 Department of Wildlife Biology, Forestry and Forest Products Research Institute, Tsukuba, Ibaraki Japan; 3 Faculty of Environmental Earth Science, Hokkaido University, Sapporo, Hokkaido Japan; 4 Biodiversity Division, National Institute for Environmental Studies, Tsukuba, Ibaraki Japan; 5 Department of Zoology, National Museum of Nature and Science Tokyo, Tsukuba, Ibaraki Japan; 6 Division of Agroecosystem Management Research, Institute for Agro-Environmental Sciences, National Agriculture and Food Research Organization, Tsukuba, Ibaraki Japan; 7 School of the Environment, The University of Queensland, Brisbane, QLD Australia; 8 Centre for Biodiversity and Conservation Science, The University of Queensland, Brisbane, QLD Australia; 9 Hokkaido Agricultural Research Center, National Agriculture and Food Research Organization, Memuro, Hokkaido Japan; 10 Division of Climate Change Mitigation Research, Institute for Agro-Environmental Sciences, National Agriculture and Food Research Organization, Tsukuba, Ibaraki Japan; 11 Department of Tourism Science, Graduate School of Urban Environmental Sciences, Tokyo Metropolitan University, Hachioji, Tokyo Japan; 12 Japan Bird Research Association, Tokyo, Japan

**Keywords:** East Asian–Australasian flyway, Long-distance migrant, Non-native species, Open-land species, Temperature niche, Waterbird species, Ecology, Ecology, Environmental sciences

## Abstract

Understanding the current status of biodiversity is crucial to preventing its loss in a changing world. We examined changes in the geographical range size and abundance of 165 bird species breeding in Japan during the past 40 years, as well as temperature niche changes in the past 20 years. Higher temperatures were recorded within the ranges of non-native species than in those of native species, and we detected range-size expansion and increased abundance among non-native species. Although open-land species exhibited range reductions from the 1970s to the 1990s, many recovered and the ranges of only a few species declined after this period. Nevertheless, the abundance of open-land species did decline, despite range-size recovery; similar inconsistencies were detected for waterbirds and raptors. Analysis of long-term temperatures suggested that species left warmest areas within their distributions while maximum temperatures experienced by species during the survey years did not change systematically. Birds in warm regions may be facing a crisis, with attrition of native bird communities and expansion of non-native species. It is necessary to establish efficient measures to prevent further expansions of non-native species and conservation measures of native species within managed areas in warm regions with few intact habitats.

**Supplementary Information:**

The online version contains supplementary material available at 10.1038/s41598-025-01382-8.

## Introduction

The recent geological history of the Earth is marked by anthropogenic impacts on the environment and accelerated biodiversity loss^1^. Biodiversity loss is not only caused by effects of individual anthropogenic imapcts but also their synegies such as habitat loss and hunting^2^ and land-use and climate change^3^. However, the trajectory of biodiversity loss exhibits wide variation among species, functional groups, and regions^4,5^, including temporal variation caused by changing threats to biodiversity^6,7^. Therefore, it is crucial to understand the current status of biodiversity and develop measures against potential threats.

Among anthrpogenic imapcts, changes in land use and climate have been suggested to be the major drivers of recent changes in species distribution and abundance^8,9^. Species in many taxa have exhibited shifts to cooler areas (i.e., higher elevations and latitudes) as well as phenology shifts^10^. Deforestation has caused the local extirpation of many species^11,12^, and agricultural intensification and farmland abandonment have induced population declines^13,14^. Besides agricultural intensification, farmland abandonment and subsequent forest maturation lead to habitat loss and degradation of early-successional or open-land species^15,16^. For migratory species, even land-use changes in non-breeding grounds can reduce breeding populations^17,18^.

**Fig. 1:**
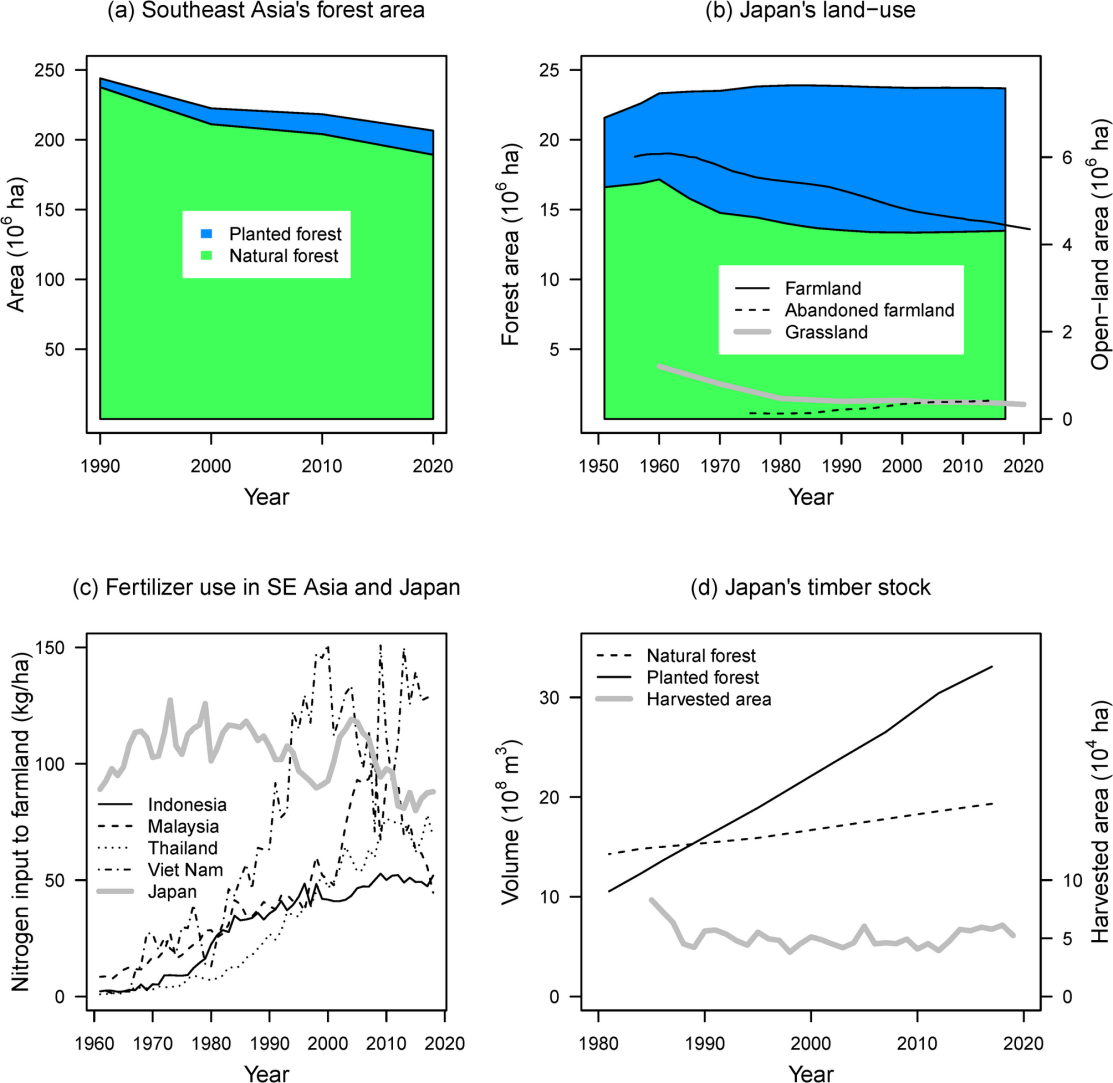
Historical land-use changes in Southeast Asia and Japan, in terms of (**a**) proportions of natural and plantation forest areas, (**b**) proportions of forest and open land, (**c**) nitrogen-based fertilizer use, and (**d**) timber harvest. In (**a**), forest-area data were obtained from a 2020 global forest-resource assessment of 10 Association of Southeast Asian Nations (ASEAN) countries^21^. In (**b**,**d**), forest-area and timber-stock data were obtained from 1954–2018 forestry statistics^22^. Abandoned-farmland and grassland area data were obtained from a long-term cumulative agriculture and forestry census^23^ and farmland area data were obtained from a cropland survey^24^. Estimated harvested areas in Japan were obtained from Shimizu and Saito^25^. In (**c**), source data were obtained from FAOSTAT.

East Asia is considered a global biodiversity hotspot due to its rich endemic biota, despite declining native vegetation^19^. While deforestation and agricultural intensification are ongoing in much of Southeast Asia, Japan has already replaced more than 40% of its native forests with tree plantations; these forests are maturing, while grasslands and farmlands are declining in area (Fig. 1). Such land use issues are important for both resident and migratory bird species because the East Asian–Australasian flyway hosts more than 40% of the global migratory bird species that breed in northern temperate/boreal regions, and these birds travel long distances to reach Southeast Asia during the non-breeding season^20^.

More than a decade ago, changes in the range sizes of Japanese breeding-bird species from the 1970s to 1990s were examined, as well as their associations with ecological traits, based on national breeding-bird surveys^17,26^. Declines in open-land species, and increases and declines in forest resident and long-distance migratory species, respectively, were found^17^. However, Japan’s forests have continued to mature, and pesticide use on farmlands has greatly decreased^27^. Since the number/amount of applied insecticide has negative impacts on farmland biodiversity^28^, decreased pesticide use is expected to restore farmland biodiversity. Meanwhile, in Southeast Asia, there were countries experiencing both increases and declines in forest areas over time^21^.

Katayama, et al.^14^ recently showed the nationwide declines of forest-generalist and open-land specialist bird species in Japan over the past 12 years. They used the data from the nationwide monitoring sites where birds were surveyed more intensively than breeding bird surveys; that is, each of 119 sites were visited up to six times during each breeding season. On the other hand, the latest breeding-bird survey in Japan recently completed and provided range-size data for three periods, the 1970s, 1990s, and 2010s with > 2,300 line transects^29,30^. In this study, we examined changes in the breeding-range sizes of Japanese birds during the past ca. 40 years and their associations with ecological traits (see Fig. S1-1 for the comparison between Katayama, et al.^14^ and ours). We also examined changes in the total numbers of detected individuals (i.e., total abundance) between the 1990s and 2010s. We adopted the species temperature index (STI) as an indicator of the temperature niche of each species, and further assessed its associations with range size and abundance. STI is a long-term average temperature experienced by species over the range, and used to examine the effects of climate change on birds^31^.

As most of non-native bird species in Taiwan increased their abundance^32^, we predicted that non-native species would have expanded their range size and abundance over time, as well as increases in the range size and total abundance of forest species due to forest maturation, increased abandoned-farmland area, and the maturation of trees planted after World War II in urban areas^33^. Predictions related to open-land bird species are difficult because increased abandoned-farmland area is expected to increase the abundance of grassland species^34^ while many of abandoned farmland and grassland likely turn into the forests for the long term. Although awareness of the need for conservation measures is increasing in East Asia, varied threats still exist^35^ and migratory birds may have decreased. Since annual and spring temperatures increased consistently during 1970–1990s and 1990–2010s in Japan^36^, we also predicted that birds tracked warming temperatures and long-term temperature within birds’ distributions declined from 1990s to 2010s.

## Methods

### Breeding-bird surveys in Japan

The Ministry of the Environment of Japan conducted breeding-bird surveys in 1978, 1997–2002, and 2016–2021. These surveys were conducted using line transects (ca. 3 km long) and questionnaires. Line transects were systematically established such that two transects were included in every 20 × 20-km grid in Japan. Transects were established to cover major habitats in the corresponding 20 × 20-km grids. Surveys were usually conducted during May to June after the arrival of migratory birds to cover the breeding bird communities, and specific dates were adjusted by the elevation and latitude based on the local knowledge.

In total, 2,336, 2,317, and 2,344 transects were surveyed in the first, second, and third surveys, respectively. The surveyors slowly (2 km/h) walked the transects and recorded all bird species detected within unlimited distance. In the latest national survey, detections were recorded separately for birds within and beyond 50 m; however, we pooled these data for abundance comparisons with the two previous surveys. Surveyors also recorded birds during 30-min watches at two points along each route; we pooled these data with those from the line transect. Questionnaire surveys were conducted to collect casual observations of the species before and after the survey dates.

All methods were carried out in accordance with relevant guidelines and regulations. Japan’s breeding bird survey was completed and the resulting data were published and made publicly available^29,37^. This study analyzed these data, and therefore, no ethical approval was required.

### Data collection and arrangement

We tallied the numbers of 20 × 20-km grid cells with detected individuals as a measure of range size for each species based on the line-transect and questionnaire-survey data. For the second and third surveys, in order to use the data from the transects that did not greatly change over time, we obtained the total abundance from 1,961 transects with ≤ 24% changes in transect lines. Among these, we selected 165 species recorded in ≥ 25 grid cells in at least one of the three surveys as the target species in our analysis. We excluded three species: the Japanese leaf warbler (*Phylloscopus xanthodryas*), spotted nutcracker (*Nucifraga caryocatactes*), and streaked shearwater (*Calonectris leucomelas*). The warbler was excluded because the latest survey adopted a different taxonomy (i.e., individuals of this species were divided into three species). One constituent species, the Kamchatka leaf warbler (*Phylloscopus examinandus*), stopovers in Japan to reach northern breeding grounds, while another species, the Japanese leaf warbler (*Phylloscopus xanthodryas*), breeds in Japan, which makes inter-survey comparisons difficult. The nutcracker was excluded because this is an alpine species and difficult to be covered by the survey. Specialized survey schemes (e.g., questionnaire survey) are required, but were not conducted in 1990s. The shearwater was excluded because it was the only seabird species. As a result, 162 species were used in subsequent analyses (Appendix S3).

We calculated ratios for the range size and total abundance in different decades on a logarithmic scale^38^, and used these as response variables in regression analysis. For example, the change in range size from the 1970s to the 1990s was calculated as $$\:\text{l}\text{o}\text{g}\left(\raisebox{1ex}{${N}_{1990s}+1$}\!\left/\:\!\raisebox{-1ex}{${N}_{1970s}+1$}\right.\right)$$, where *N*_1990s_ and *N*_1970s_ are the range sizes for each decade; we added 1 to both the numerator and denominator to avoid zero values. When obtaining the total abundance, we alleviated the effects of outliers (i.e., exceptionally high site-level values) by replacing values higher than the 95% percentile with the 95% percentile. This was because there were large flocks for varied species (e.g., great cormorant *Phalacrocorax carbo*, black kite *Milvus migrans*, tree sparrow *Passer montanus*), which has great impacts on the total abundance.

Among the 162 species, we compared STI values and range size and total abundance changes between 6 non-native and 156 native species. STI was calculated as the abundance-weighted median value of annual (mean) temperature (ATP) in Japan, following Ueta, et al.^39^. This index was calculated based on data from the latest breeding-bird survey, which was also analyzed in this study; however, we do not consider that this approach had any major impact on our results.

### ATP of survey transects

To characterize the distribution of each bird species, we calculated the 30-year average (1981–2010) ATP for each transect, which was strongly correlated with the mean temperature during the breeding season (May–July; *r* = 0.98). ATP values for each distribution were obtained using the same method used to obtain STI. Thus, we used the ATP values at 1-km resolution (Mesh Climatic Data 2010, provided by the Japan Meteorological Agency), with overlapping individual transects (each transect was usually larger than a 1-km grid), and averaged the ATP values. Next, ATP values were extracted from the transects in which each species was detected, and the number of the ATP value was multiplied by the species abundance for each transects. We obtained minimum, median, and maximum abundance-based ATP values of the distribution for each species during the 1990s and 2010s.

To evaluate the changes in ATP experienced by each species during the past 20 years, we created a new spatial grid of ATP data with 1 km resolution for each survey year within the 1990s and 2010s. Survey-year ATP was derived from daily meteorological observation data obtained by Automated Meteorological Data Acquisition System stations and the Mesh Climatic Data. We applied the spatial interpolation method developed by Seino^40^ and Ishigooka, et al.^41^. We averaged the ATP values among survey years to obtain the short-term, survey-year ATP for each transect, and then calculated the minimum, median, and maximum ATP values for the 1990s and 2010s for each species.

### Regression analysis

We examined the effects of ecological traits (described below) on changes in range size (1970–1990s, 1990–2010s, and 1970–2010s), total abundance (1990–2010s), STI, and 30- and survey-year ATP during the past 20 years using ordinary least-squares (OLS) regression analysis. ATP changes were calculated in terms of minimum, median, and maximum values. To evaluate the effects of ecological traits across different measures, we applied a model averaging using the “model.avg” function in the *MuMIn* v1.47.1 R package^42^. We evaluated all models (subset = TRUE) based on the small-sample version of the Akaike information criterion (AICc), while we averaged the coefficients over the models where the parameters appeared (‘conditional’ average). This is because an alternative average method (‘full’ average) can undervalue the coefficients and associated significant levels^43^. We then examined the parameter estimates of the models with the lowest AICc values to assess the graphical relationships between the response variables and ecological traits.

To consider phylogenetic dependence among species, we also constructed phylogenetic generalized linear models with the same explanatory variables included in the best OLS regression models. We used the “pgls” function in the *caper* v1.0.2 R package^44^ and created two models to deal with one (λ) and three parameters (λ, δ, κ), respectively, as free parameters, and compared the estimates with those obtained from OLS regression. We obtained 95% confidence intervals (CIs) of the coefficients by multiplying the standard error (SE) by 1.96. The phylogenies of 156 target species were obtained from BirdTree.org^45^ based on the Hackett backbone (Stage2 Mayr All Hackett), and single consensus tree with average edge length was obtained using the “consensus.edges” function of the *phytools* v1.2-0 R package^46^. We excluded absent edges from the computation of average edge length. We selected the Hackett backbone because the analysis of 19 independent loci by Hackett, et al.^47^ appears to have been more reliable than that of five loci including β-fibrinogen combined with fossil records by Ericson, et al.^48^. Recent genomic analyses broadly support the Hackett phylogeny^49,50^ and β-fibrinogen may bias the phylogeny of Ericson^51^. We excluded two species (Japanese quail, *Coturnix japonica*, and yellow-breasted bunting, *Emberiza aureola*) from our analyses of temperature changes in the range distribution, because they were not detected in either of the two recent surveys and corresponding temperatures were not available.

We considered nine ecological traits in the analysis based on our previous studies^17,26^: habitat type, hand–wing index (HWI), body weight (g), range size, migration type (resident, short- or long-distance migration), productivity (possible number of juveniles produced per year), STI, use of farmland and urban areas (both are binary variables). We included farmland and urban area use because this ability can influence species distribution and therefore range and abundance dynamics via adaption to these human-made habitats^26^. We did not detect strong correlations among continuous trait variables (|*r*| < 0.39) or between categorical variables and others. We did not use the number of habitat categories as an index of habitat specialization because this parameter was strongly correlated with farmland use. Habitat type was a categorical variable with five classes: forest, open land, raptor, river, and water. Open-land species comprised birds that utilize bare ground and grasslands. Raptor was designated as a separate habitat category because raptors typically use a combination of habitats, e.g., nesting in forests and foraging in surrounding open areas^17^. River and waterbird species were considered separate categories to evaluate differences between environments with running and still water. HWI data, which represent wing shape and dispersal ability, were obtained from Sheard, et al.^52^. The range size trait refers to the largest range size among the three surveys. We logarithmically transformed body weight and range size data to mitigate the effects of outliers, and we used the quadratic term of body weight to consider nonlinear effects. We also conducted the same regression analysis for forest species alone; in this case, we did not use habitat type but rather foraging type (canopy, flycatcher, ground, omnivore, shrub, or stem) and nesting substrate (canopy, ground, shrub, or tree). Other trait values were obtained from publications^17,26,53^. We excluded the white-throated needletail (*Hirundapus caudacutus*) from our analysis of forest species because it was the only species in the dataset that conducts aerial foraging.

In our regression analysis, we gave individual species equal weight, and did not consider differences in uncertainties among species because the “pgls” function cannot consider weights. Therefore, we examined the effects of considering weights using OLS with STI as a response variable. We expected that range size would cause uncertainty, such that STI values of rare species would be less reliable than those of common species. First, we regressed STI without explanatory variables, with only an intercept in the model, to obtain the residuals. Then the absolute values of the residuals were regressed by the log-transformed range size, and this model was used to represent the variance of uncertainties for individual species as a function of range size. We constructed the OLS model using the inverse of the variances (residuals) as weights and compared the results with those of OLS without weights. The model selection tables and parameter estimates were similar (Table S2-1). Therefore, we did not consider regression weights in our regression models.

## Results

### Non-native species

**Fig. 2:**
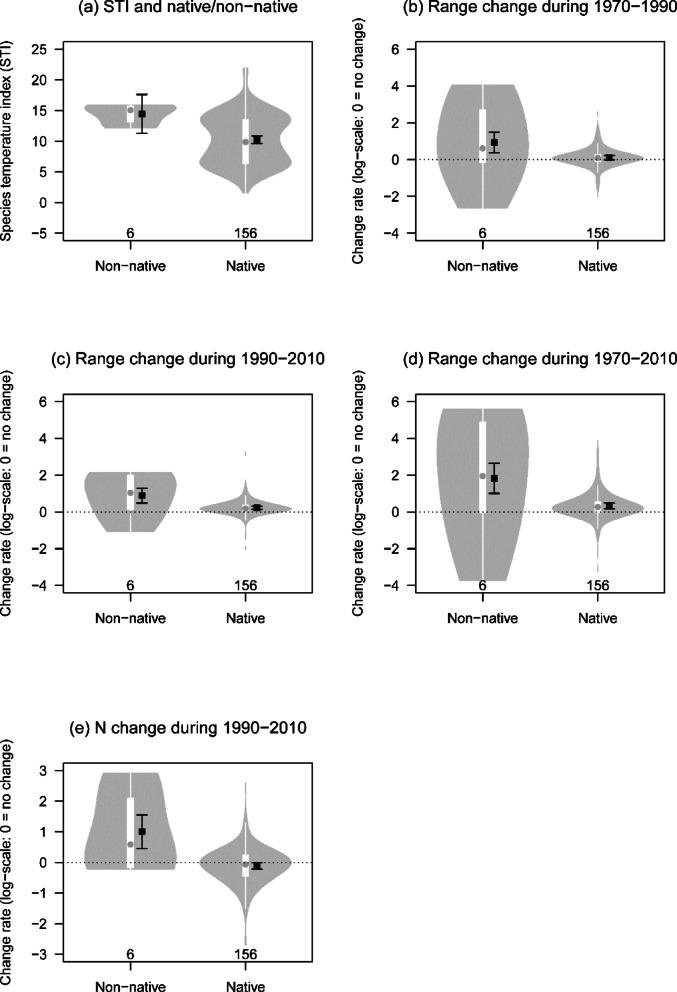
STI, range and abundance (N) change in relation to non-native/native species. Numbers of species in each group are shown at the bottom of the plot; mean estimates and their 95% CIs, which were obtained based on the OLS model by omitting the intercept in the “lm” function in R (cell-means model), are aligned next to violin plots. Violin plots are depicted by “vioplot” ver. 0.5.0^54^ where interquartiles, median, lower and upper whiskers are shown in the plots.

Non-native species had distinct characteristics compared to native species, with higher STIs and increased range size and abundance during the past 40 years (Fig. 2). There were two exceptions, the red avadavat (*Amandava amandava*) and Chinese bamboo partridge (*Bambusicola thoracicus*) (log-scale change rate in range size during the 40 years: red avadavat [−3.76], Chinese bamboo partridge [−0.16]: Appendix S3). The statuses of the red-billed leiothrix (*Leiothrix lutea*) and Chinese hwamei (*Garrulax canorus*) were particularly remarkable, as their ranges had expanded more than three-fold during the past 20 years (Appendix S3).

### STI comparisons

**Fig. 3:**
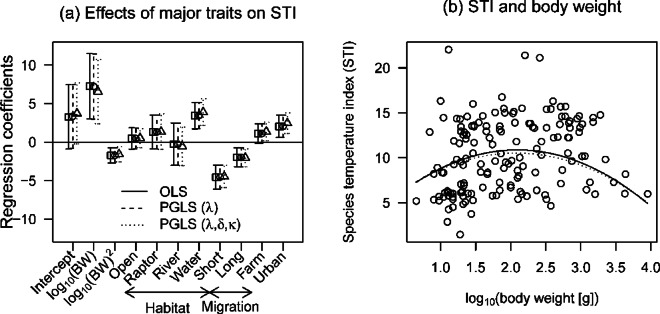
(**a**) Effects of major ecological traits on the species temperature index (STI) and (**b**) estimated effects of body weight on STI for breeding birds in Japan. Data shown in (**a**) are parameter estimates for the best ordinary least-squares (OLS) model. Two phylogenetic regression models with the same trait variables were fitted. Forest and resident species are reference categories in the habitat and migration groups, respectively.

Our analysis excluding non-native species showed that forest and open-land species had lower STIs and waterbird species had higher STIs (Fig. S2-1a). Among migration types, resident and short-distance migratory species had higher and lower STIs, respectively (Figs. 3a, S2-1b, Table S2-3). Nonlinear body-weight effects were caused by low STIs among many light-weight species and the lack of high STIs for heavy-weight species (> 2,000 g), such as the red-crowned crane (*Grus japonensis*) and golden eagle (*Aquila chrysaetos*) (Fig. 3b). All phylogenetic regression models constructed in this study yielded similar estimates based on OLS regression, as the λ parameter was estimated to be close to zero.

## Changes in range size and total abundance

**Fig. 4:**
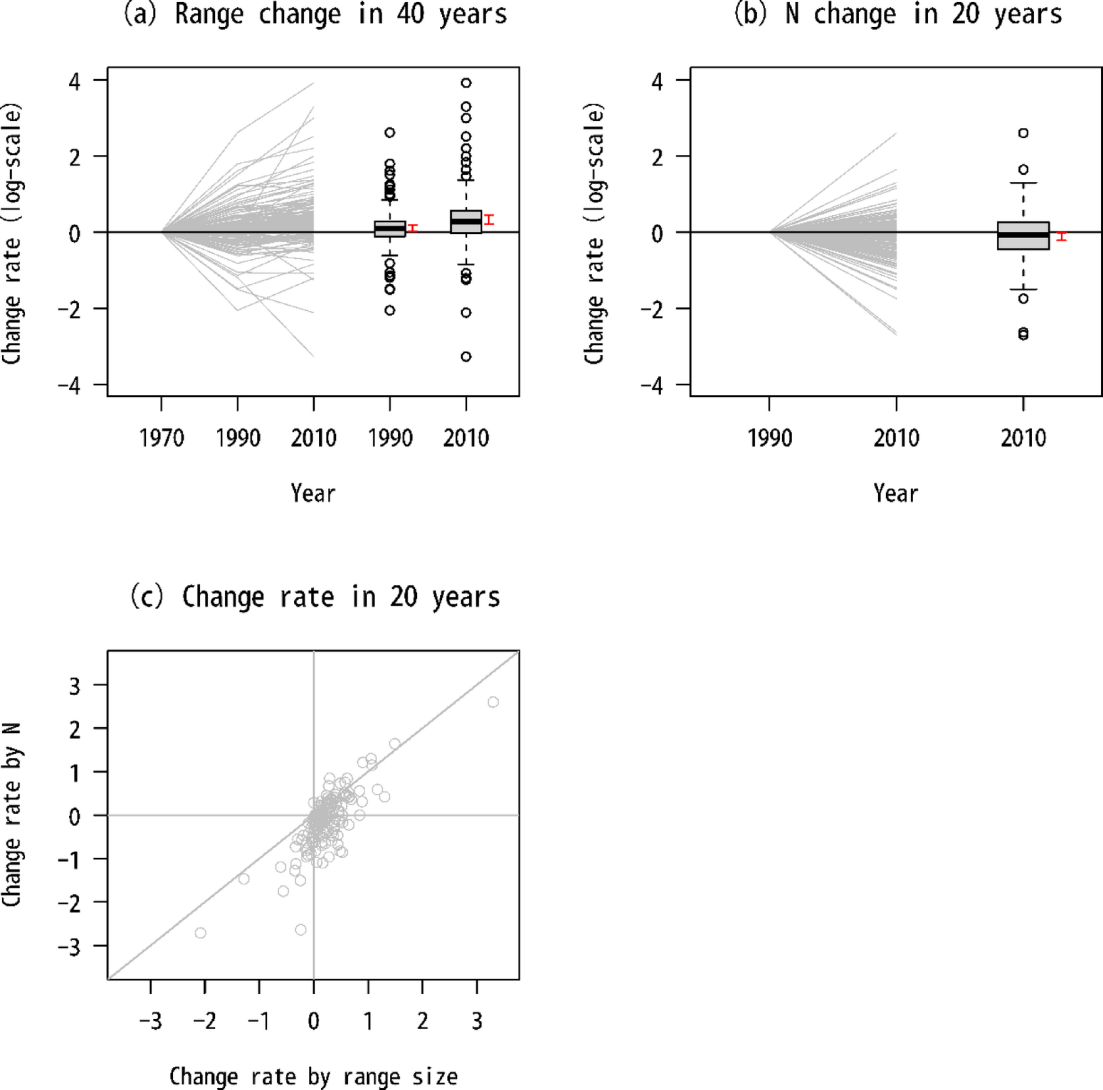
Changes in the range and abundance (N) of Japanese birds during the past 40 years, calculated as ratios between different decades. (**a**) Ratio of range size in the 1990s and 2010s to the 1970s for each species examined in this study. Ratio of (**b**) abundance and (**c**) range size and abundance in the 2010s to the 1990s. Red vertical bars next to the box plots indicate 95% confidence intervals (CIs) of mean values obtained from OLS regression with only an intercept.

**Fig. 5:**
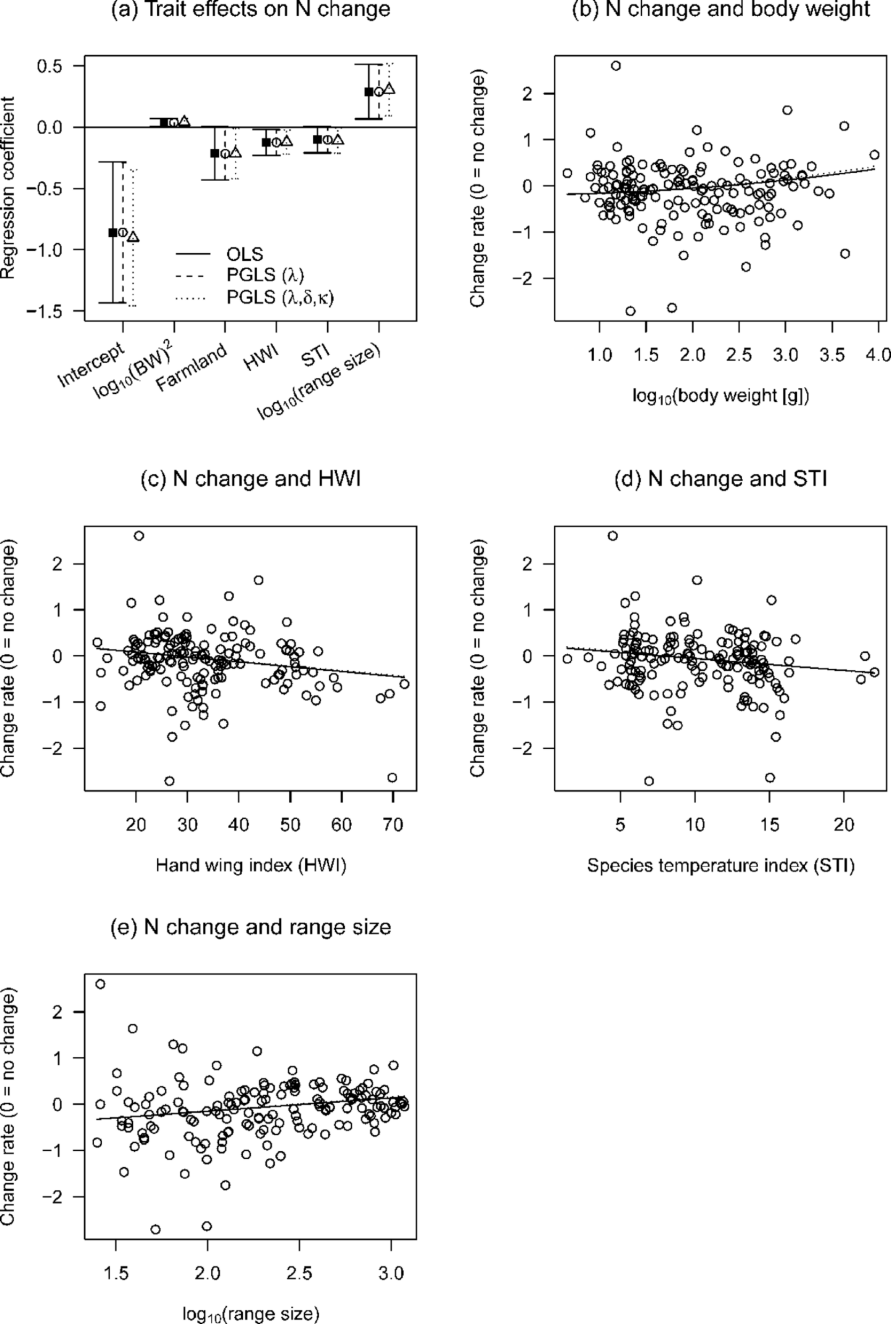
Effects of ecological traits on changes in species abundance from the 1990s to 2010s. Regression coefficients for each trait (a), Estimated effects of body weight (BW) (**b**), hand–wing index (HWI) (**c**), STI (**d**), and range size (**e**). Data used in (**a**) are parameter estimates for the best OLS model.HWI and STI data were standardized prior to analysis for easier comparison.

Although the species analyzed in this study generally expanded their ranges over the past 40 years (Fig. 4), there were two major patterns in range-size change among species. One showed continued increases throughout the past 40 years, and the other showed a decrease from the 1970s to 1990s and an increase from the 1990s to 2010s. Open-land species showed range-size recovery in the 2010s and only two species, the yellow-breasted bunting and brown shrike (*Lanius cristatus*), showed clear continued declines (log-scale change rage in range size during 1990–2010: yellow-breasted bunting [−2.08], brown shrike [−0.61], Fig. S2-2b). However, abundance increased to a smaller degree than range size, with some species exhibiting decreased abundance irrespective of range-size increases (Fig. 4). This opposing trend was evident for open-land, raptor, and waterbird species (Fig. S2-2b, d). Ecological traits were associated with changes in range size from the 1970s to 1990s; however, such associations were not detected from the 1990s to 2010s (Table 1). The amount of variation in abundance change among species was not explained by habitat type but rather by other traits such as HWI and range size (Fig. 5).

**Table 1:** Effects of ecological traits on distribution changes for breeding-bird species in Japan.

Trait	Range and abundance (*N*) change					Temperature					
	Range change					30-year average change ^e^			Survey-year average change ^e^		
	~ 1990	~ 2010	40years	N (~ 2010)	STI	Min	Median	Max	Min	Median	Max
Habitat: Open ^a^							+++			+++	
Raptor ^a^							++		++	++	
River ^a^							+			+	
Water ^a^				---	+++				+	++	
Hand–wing index				-		++		-	+		
log_10_(body weight)	---				+++						
log_10_(body weight)^2^	+++		+		---						
log_10_(range size)	+			+			+		++		
Migration: Short ^b^					---			--			
Long ^b^	--		--		---			--			
Productivity								++			+
STI ^c^					NA		--		---	---	
Farmland ^d^				--							
Urban ^d^					+++						

Effects of nine traits (two categorical) were quantified and compared by the model average. Signs of parameters with low significance (up to *p* < 0.1) are shown: *p* < 0.1: +, *p* < 0.05: ++, *p* < 0.01: +++. Negative signs follow the same rule.

^a, b^ Reference categories: habitat (forest), migration (resident). ^c^ Species temperature index. ^d^ Binary indicator variables describing whether species occur at farmland/urban or not. ^e^ Minimum, median, and maximum annual temperature of distributions for 30- and survey-year means were obtained, and associations of their differences between the 1990s and 2010s with ecological traits were examined.

Among individual habitat groups, body weight had positive effects on abundance for waterbird species (Table S2-2), implying that species with declined abundance were lighter-weight species (Fig. S2-7e). Although migration type poorly explained interspecific variation in abundance changes, long-distance migratory species exhibited declined abundance for waterbirds and open-land species in general (Fig. S2-7b, c). Forest species exhibited increased abundance of long-distance migrants (Fig. S2-7d), whereas among breeding birds as a whole, long-distance migratory species showed abundance declines throughout the past 20 years (Fig. S2-7a).

**Fig. 6:**
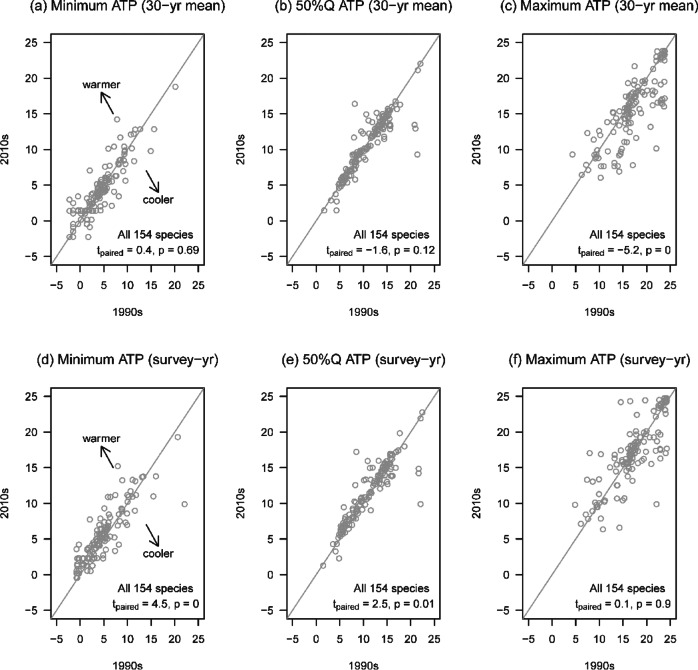
Shifts in species distributions in response to annual mean temperature. (**a**–**c**) Long-term (30-year average) annual mean temperatures were obtained for each transect, and minimum (**a**), median (**b**), and maximum (**c**) temperatures within species distributions were compared between the 1990s and 2010s. If a species did not change its distribution in association with long-term temperature, then its data point appears on the 1:1 line. (**d**–**f**) Annual temperatures were obtained from years when field surveys were conducted; minimum (**d**), median (**e**), and maximum (**f**) temperatures are compared. Since the survey-year temperature generally became warmer throughout Japan (Fig. S7f), points lying along the 1:1 line imply that the species retained the same experienced temperature despite the warming temperature; thus, such species shifted their distributions towards warmer areas. The results of paired *t*-tests comparing annual temperatures of individual species are shown in the panels. Associated *p*-values were rounded to two digits.

**Fig. 7:**
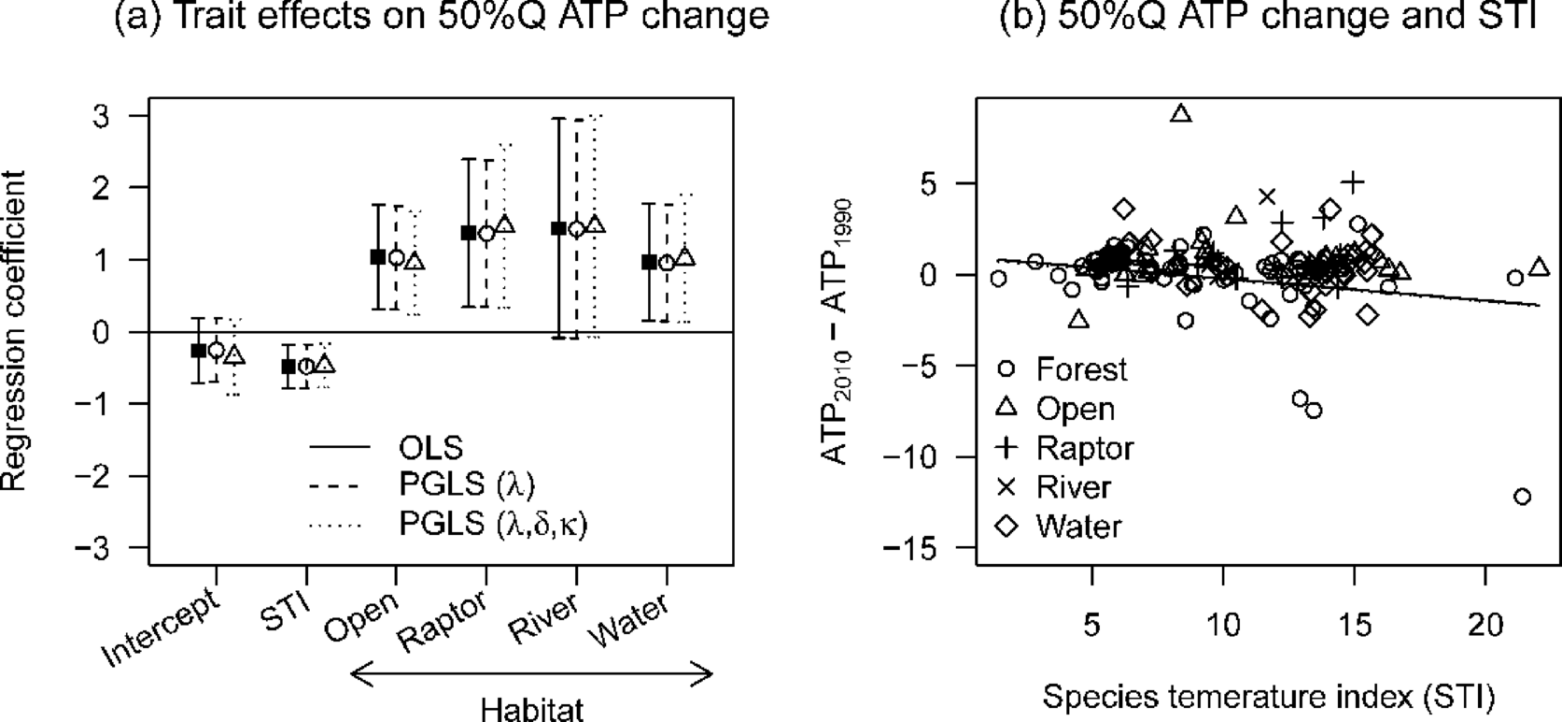
Effects of ecological traits on changes in survey-year annual mean temperature within species distributions. (**a**) Regression coefficients for STI and four habitat categories: open land, raptor, river, and water (see text for category explanations). (b) Estimated effects of STI. Data in (**a**) are parameter estimates of the best OLS model. STI was standardized prior to analysis for easier comparison. Median annual mean temperature was obtained for each species in the two latest surveys, and their differences were calculated (ATP_2010s_ – ATP_1990s_) and analyzed through regression analysis.

## Changes in temperature niches

Our analysis of long-term annual mean temperature within distributions showed that maximum temperature has declined over the past 20 years (Fig. 6c), implying that bird species generally left warm areas (Fig. S2-8). However, this systematic change was not observed in minimum and median temperatures (Fig. 6a, b). Strong trait associations with these changes were not observed (Table 1). Survey-year annual mean temperature showed that transects experienced higher temperatures across Japan over time (Fig. S2-5f). Differences in survey-year temperatures over the past 20 years also showed that minimum and median temperatures became warmer (Fig. 6d, e). This trend was observed for all habitat groups except forest species (Table 1; Figs. 7a, S2-6). The opposite pattern was observed for species preferring higher temperatures.

## Discussion

### Changes in range size, abundance, and temperature

Japanese breeding-bird species generally expanded their ranges during the past 20 years. However, these changes did not necessarily reflect abundance changes. Many species with restored range sizes exhibited decreased abundance, and changes in range and abundance may have been influenced by different drivers. For example, decreased pesticide use^27^, forest maturation^17^, and increases in abandoned farmland area^34^ (Fig. 1) likely have contributed to the expansion of marginal habitats among various species. However, there are threats that can degrade primary habitats, such as grassland degradation and climate change. Thus, it is important to record abundance for broad-scale species monitoring.

We found decreased long-term maximum temperatures within distributions in the past 20 years but not in maximum temperatures specific to survey years (Fig. 6f), suggesting that breeding bird species tracked warming temperatures in warm areas. However, these patterns were not observed in minimum temperatures. There are few areas with annual mean temperatures cooler than 5 °C in Japan, and in southern Japan (excluding Hokkaido), few areas are cooler than 7.5 °C (Fig. S2-9). Thus, migration (distributional shift) into cooler areas are restricted by geography. Therefore, the distribution and abundance of forest and open-land species preferring cool areas may further decline in the future, as is concerned in the European Alps^55^. Birds leaving warm areas may also have been caused by smaller population sizes in southern Japan due to fewer intact areas (see *Conservation implications*).

Range shifts towards cooler areas were observed for species with high STIs (Table 1; Fig. 7); the three species showing the largest declines in survey-year median annual temperature were the Japanese scops owl (*Otus semitorques*), Japanese paradise flycatcher (*Terpsiphone atrocaudata*), and ruddy kingfisher (*Halcyon coromanda*). These forest species likely expanded into mature forests under warming temperatures, as a rare reported case of habitat-specific climate tracking. In contrast, the ranges of species belonging to other habitat groups became warmer (Table 1; Figs. 7a, S2-6), implying that they may not have been able to track climate changes. The dispersal of these species may be restricted by unsuitable habitats, as Japan is a forested country (67% of the total land cover). Alternatively, they may have a wide temperature niche and be able to resist warming temperatures, although climate tracking may be detected in future studies due to lower reproductive success in open habitats in hot years^56^.

### Decline of open-land and waterbird species

Declining abundance of open-land species would be related to decreases in farmland and grassland areas (Fig. 1). Declines in waterbird species were unexpected, which also may indicate the degradation of their habitats. For example, although pesticide use has been greatly reduced in recent decades^27^, compositional changes in agrochemicals, such as the recent application of neonicotinoids, may have decreased their abundance directly/indirectly^57,58^. Light-weight waterbird species have exhibited abundance declines, and larger species may not be exposed to threats such as predation pressure^59^. Notably, the abundances of long-distance migratory waterbird and open-land species declined, whereas that of forest species did not, perhaps due to strong hunting pressure in open habitats and agricultural intensification in non-breeding grounds of Southeast Asia^35,60^.

### Dynamics of forest species

Using annual population count data collected across Japan, Katayama, et al.^14^ found increased abundance of forest-specialist species. In this study, contrary to our predictions, we did not observe a clear overall increase in the abundance of forest species (Fig S2-2d); however, ground- and cavity-nesting, and stem probing species exhibited a general increase (Fig. S2-4). Thus, although Japan’s forests are becoming more mature, certain threats have caused declines in some forest species. We were unable to identify the leading traits associated with these declines; therefore, it is necessary to monitor species such as the brown-headed thrush (*Turdus chrysolaus*), marsh tit (*Poecile palustris*), and dark-sided flycatcher (*Muscicapa sibirica*) to determine whether they will continue to decline in abundance.

### STIs and ecological traits

The mean STI values of forest and open-land species were lower than those of other habitat groups (Figs. 3a, S2-1a). Forests and grasslands located in cooler areas such as deciduous forests are productive, attractive breeding habitats for birds^61^. Japan’s once vast wetlands now remain only in northern areas^62^. In this study, short-distance migrants had lower STI values compared to resident and long-distance migrants (Figs. 3a, S2-1b), which may avoid competition with other groups. Waterbird species had the highest STI values among all habitat groups (Figs. 3a, S2-1a), likely because warm areas of southern Japan harbor rich invertebrate biotas as potential food resources^63^. Farmlands, particularly rice paddies, are also heavily distributed in warmer areas of Japan^64^.

### Limitations and future directions

Our nationwide analysis primarily focused on the extent of the changes occurring in Japanese breeding birds. Future studies need to investigate the specific mechanisms driving these large-scale distributional changes. For example, we hypothesize that certain land-use features, such as increased abandoned farmland, may facilitate population colonization of forest species or lead to local extinction of bare-ground species at their range margins^34^. Long-term monitoring programs could help identify specific climatic events, such as extreme heat, that influence population dynamics in these areas^56^. Additionally, inconsistencies between range expansions and population declines might be attributable to low reproductive success at the range margins. Future studies also need to focus on spatial predictions of future expansions of non-native species^65^ and range shifts in cool-temperature-adapted species^55^, and the identification of environmental conditions in microrefugia^66^. These efforts are crucial for mitigating the impacts of human activities. Moreover, with future climate change, the conservation value of existing protected areas may change, potentially compromising their capacity to ensure population persistence^67^.

### Conservation implications

Bird communities in warm regions of Japan may be facing a crisis due to the retreat of native species and alarming expansion of non-native species. Japan is an island country surrounded by the ocean, and species colonization from the south in association with warming temperatures is not common. Thus, native species may be suffering from biotic attrition^68^, as also observed in a banding survey conducted in a forest in western Shikoku, southern Japan, which captured 413 individuals in 2021^69^. Among these, a single non-native species, the red-billed leiothrix, made up 75% of the individuals. Most non-native species are introduced as captive pets and originally distributed in (sub)tropical areas; warmer temperatures are more suitable for their colonization. As non-native species are likely to expand their ranges further, and may have various impacts on native ecosystems^70^, it is necessary to study the ecology of non-native species, heighten public awareness, and establish efficient measures to prevent further expansions^71^.

Mitigating the impacts of climate change through local conservation measures is a significant challenge. Warm areas of Japan have been subject to anthropogenic modifications for 2000 years^72^, leading to fewer intact habitats such as old-growth forests^73^ and nature reserves^74^. Various species can occur in managed areas depending on local habitat structures; therefore, it is important to establish conservation measures within managed areas, such as retention forestry^75^ and organic farming^76^. Abandoned farmland is useful for wetland- and forest-species conservation^34^, and the maintenance and restoration of semi-natural grasslands are pressing issues^77^. Forestry and agriculture may play some roles in the areas surrounding remnant grasslands, such as providing temporary habitat^78^. Finally, collaborative work within the East Asian–Australasian flyway^20^ is essential, as long-distance migratory species are still declining in Japan.

## Electronic supplementary material

Below is the link to the electronic supplementary material.

Supplementary Material 1

Supplementary Material 2

## Data Availability

Source data for this study is available within the supplementary files.
