# Supplementary material for: Range size and abundance dynamics of Japanese breeding birds over 40 years suggest a potential crisis in warm areas
Source: Sci Rep. 2025 May 19;15:17281. doi: 10.1038/s41598-025-01382-8 (PMC12089301; doi:10.1038/s41598-025-01382-8)
Supplement: Supplementary file 2 — Supplementary Material 2 [file 41598_2025_1382_MOESM2_ESM.docx]

Supplementary Materials for the following manuscript:

Title:

Recovery or decline: Range size and abundance dynamics of breeding birds in Japan under climate and land use changes

Authors:

Yuichi Yamaura, Kazuhiro Kawamura, Masayuki Senzaki, Munehiro Kitazawa, Isao Nishiumi, Naoki Katayama, Tatsuya Amano, Yasushi Ishigooka, Shigeto Sudo, Takeshi Osawa, Mutsuyuki Ueta

This file includes the following materials:

- Appendix S1. Abundance change rates between this study and Katayama et al. (2024).
- Appendix S2. Detailed results of regression analysis examining dynamics of Japanese breeding birds in the past 40 years.

Appendix S1. Abundance change rates between this study and Katayama et al. (2024).


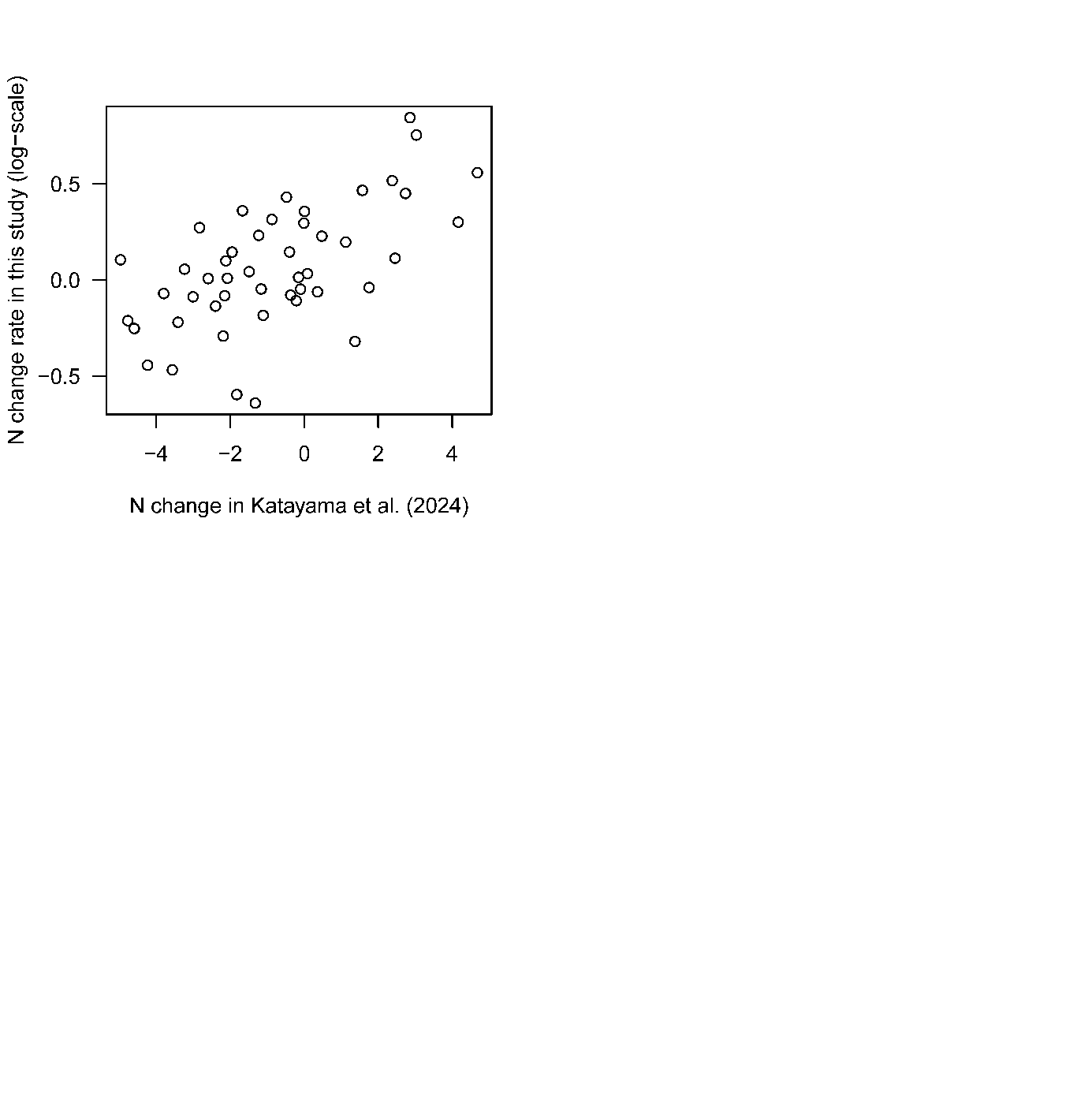


Fig. S1-1. Comparison of abundance change rates among common 47 species between this study and Katayama et al. (2024).

We calculated the change rate at log-scale (i.e., log[*N*_1990_/*N*_2010_]). Katayama et al. (2024) fitted OLS regression against log-transformed annual abundance, and took the regression slope as a measure of population trend. Therefore, the slope measures the differences in log-transformed abundance across years, which is comparable to our change rate at log-scale.

Reference

Katayama, N., Fujita, T., Ueta, M., Morelli, F., Amano, T., 2024. Effects of human depopulation and warming climate on bird populations in Japan. Conservation Biology 38, e14175.

Appendix S2. Detailed results of regression analysis examining dynamics of Japanese breeding birds in the past 40 years.

Table S2-1. Top ordinary least-squares models (Δ < 2) using STI as a response variable with and without species weights.

| (a) Unweighted model | | | | | | | | | | | | |
| --- | --- | --- | --- | --- | --- | --- | --- | --- | --- | --- | --- | --- |
| (Intercept) | Farm | Habitat | HWI | Migration | Prod | Urban | log_10_(BW) | (log_10_(BW))^2^ | log_10_(RS) | df | AICc | ΔAICc |
| 3.27 | 1.11 | + | NA | + | NA | 2.04 | 7.25 | -1.73 | NA | 12 | 814.66 | 0.00 |
| 1.01 | 0.98 | + | NA | + | NA | 1.94 | 7.29 | -1.71 | 0.85 | 13 | 815.10 | 0.44 |
| -0.08 | NA | + | NA | + | NA | 2.22 | 8.17 | -1.93 | 1.00 | 12 | 815.16 | 0.50 |
| 2.46 | NA | + | NA | + | NA | 2.39 | 8.28 | -1.99 | NA | 11 | 815.51 | 0.85 |
|  |  |  |  |  |  |  |  |  |  |  |  |  |
| (b) Weighted model | | | | | | | | | | | | |
| (Intercept) | Farm | Habitat | HWI | Migration | Prod | Urban | log_10_(BW) | (log_10_(BW))^2^ | log_10_(RS) | df | AICc | ΔAICc |
| 0.13 | NA | + | NA | + | NA | 2.26 | 6.82 | -1.62 | 1.35 | 12 | 796.92 | 0.00 |
| 1.06 | 0.79 | + | NA | + | NA | 2.05 | 6.14 | -1.46 | 1.20 | 13 | 797.51 | 0.59 |
| -0.28 | NA | + | -0.19 | + | NA | 2.36 | 7.13 | -1.68 | 1.31 | 13 | 798.85 | 1.93 |

Symbols: +, focal categorical variable was included in the corresponding model; NA, variable not included in the model.

Table S2-2. Top OLS regression model-selection table explaining abundance change rates of individual habitat groups.

| Group | Intercept | log_10_ (BW) | log_10_ (RS) | HWI | Migration | Productivity | Farmland | Urban | DF | AICc | Δ | Weight |
| --- | --- | --- | --- | --- | --- | --- | --- | --- | --- | --- | --- | --- |
| Waterbirds | −1.92 | 0.59 | NA | NA | NA | NA | NA | NA | 3 | 74.85 | 0.00 | 0.15 |
|  | −2.22 | 0.61 | NA | NA | NA | 0.04 | NA | NA | 4 | 76.21 | 1.36 | 0.08 |
|  | −2.03 | 0.65 | NA | NA | NA | NA | NA | −0.84 | 4 | 76.22 | 1.37 | 0.08 |
| Open-land | −0.16 | NA | NA | NA | NA | NA | NA | NA | 2 | 91.82 | 0.00 | 0.07 |
|  | 0.21 | NA | NA | −0.01 | NA | NA | NA | NA | 3 | 92.30 | 0.47 | 0.05 |
|  | −0.22 | 0.38 | NA | −0.02 | NA | NA | NA | 0.45 | 5 | 92.68 | 0.86 | 0.04 |
|  | −0.15 | 0.29 | NA | −0.01 | NA | NA | NA | NA | 4 | 92.72 | 0.89 | 0.04 |
|  | −0.15 | NA | NA | NA | + | NA | NA | NA | 4 | 92.91 | 1.09 | 0.04 |
|  | −0.48 | 0.20 | NA | NA | NA | NA | NA | NA | 3 | 93.13 | 1.31 | 0.04 |
|  | 0.24 | NA | NA | −0.01 | NA | NA | NA | 0.31 | 4 | 93.52 | 1.70 | 0.03 |
|  | −0.67 | 0.28 | NA | NA | + | NA | NA | NA | 5 | 93.72 | 1.89 | 0.03 |
|  | −0.32 | NA | NA | NA | NA | 0.02 | NA | NA | 3 | 93.79 | 1.97 | 0.03 |
|  | −0.08 | NA | NA | NA | NA | NA | −0.14 | NA | 3 | 93.80 | 1.98 | 0.03 |
| Forest | −0.57 | NA | 0.28 | NA | NA | NA | −0.36 | NA | 4 | 82.63 | 0.00 | 0.12 |
|  | −0.63 | NA | 0.30 | NA | NA | NA | NA | −0.24 | 4 | 84.20 | 1.58 | 0.05 |
|  | −0.62 | NA | 0.30 | NA | NA | NA | −0.29 | −0.13 | 5 | 84.34 | 1.72 | 0.05 |
|  | −0.43 | NA | 0.27 | 0.00 | NA | NA | −0.37 | NA | 5 | 84.52 | 1.89 | 0.05 |
|  | −0.52 | NA | 0.24 | NA | NA | NA | NA | NA | 3 | 84.56 | 1.94 | 0.05 |
|  | −0.72 | 0.06 | 0.30 | NA | NA | NA | −0.40 | NA | 5 | 84.57 | 1.95 | 0.05 |

OLS regression models were constructed to assess associations between ecological traits and abundance change rates for three major habitat groups, with possible combinations of seven trait covariates. Competing models with Δ < 2 are shown. We did not consider the quadratic term of body weight because scatter plots did not suggest nonlinear effects. Symbols: +, focal categorical variable was included in the corresponding model; NA, variable was not included in the model.

Table S2-3. Effects of ecological traits on distributional changes for forest species in Japan.

|  | Range and abundance (N) change | | | |  |  | Temperature | | | | | | |
| --- | --- | --- | --- | --- | --- | --- | --- | --- | --- | --- | --- | --- | --- |
|  | Range change | | |  |  |  | 30-year average ^e^ | | |  | Survey-year average ^e^ | | |
| Trait | ~1990 | ~2010 | 40 years | N (~2010) |  | STI | Min | Median | Max |  | Min | Median | Max |
| Forage: Flycatcher ^a^ | --- |  |  |  |  |  |  |  |  |  | - | - |  |
| Ground ^a^ |  |  |  |  |  |  |  | - |  |  |  | -- |  |
| Omnivore ^a^ |  |  |  | - |  |  |  |  |  |  |  |  |  |
| Shrub ^a^ |  |  |  |  |  |  |  |  |  |  |  |  |  |
| Stem ^a^ |  | +++ |  | + |  | - |  |  |  |  |  |  |  |
| Nest: Ground ^b^ |  |  |  |  |  |  |  |  |  |  |  |  |  |
| Shrub ^b^ |  |  |  |  |  |  |  |  |  |  |  |  |  |
| Tree ^b^ |  |  |  |  |  |  |  |  |  |  |  |  |  |
| Hand–wing index |  |  |  |  |  |  |  |  | -- |  |  |  |  |
| log_10_(body weight) |  |  |  |  |  |  |  |  |  |  |  |  |  |
| log_10_(body weight)^2^ |  |  |  |  |  |  |  |  |  |  |  |  |  |
| log_10_(range size) |  |  |  | ++ |  |  |  | ++ | +++ |  | ++ | ++ |  |
| Migration: Short |  |  |  |  |  | --- |  |  |  |  |  |  |  |
| Long |  |  |  |  |  |  |  |  |  |  | ++ |  |  |
| Productivity |  |  |  |  |  |  |  |  |  |  |  |  |  |
| STI ^c^ | + |  | + |  |  | NA |  | --- |  |  | --- | --- |  |
| Farmland ^d^ | - |  | -- | - |  |  |  |  |  |  |  |  |  |
| Urban |  | - |  |  |  | ++ |  |  |  |  | ++ |  |  |

Effects of nine traits (three categorical) were quantified and compared by model averaging. Signs of parameters with low significance (up to *p* < 0.1) are shown: *p* < 0.1: +, *p* < 0.05: ++, *p* < 0.01: +++. Negative signs follow the same rule.

^a, b^ Canopy forager is a reference foraging category; cavity is a reference nesting category.

^c^ Species temperature index. ^d^ Binary indicator variables describing whether species occur in farmland or urban areas. ^e^ Minimum, median, and maximum annual temperature of distributions for 30- and survey-year means were obtained, and associations with their differences between the 1990s and 2010s with traits were examined.


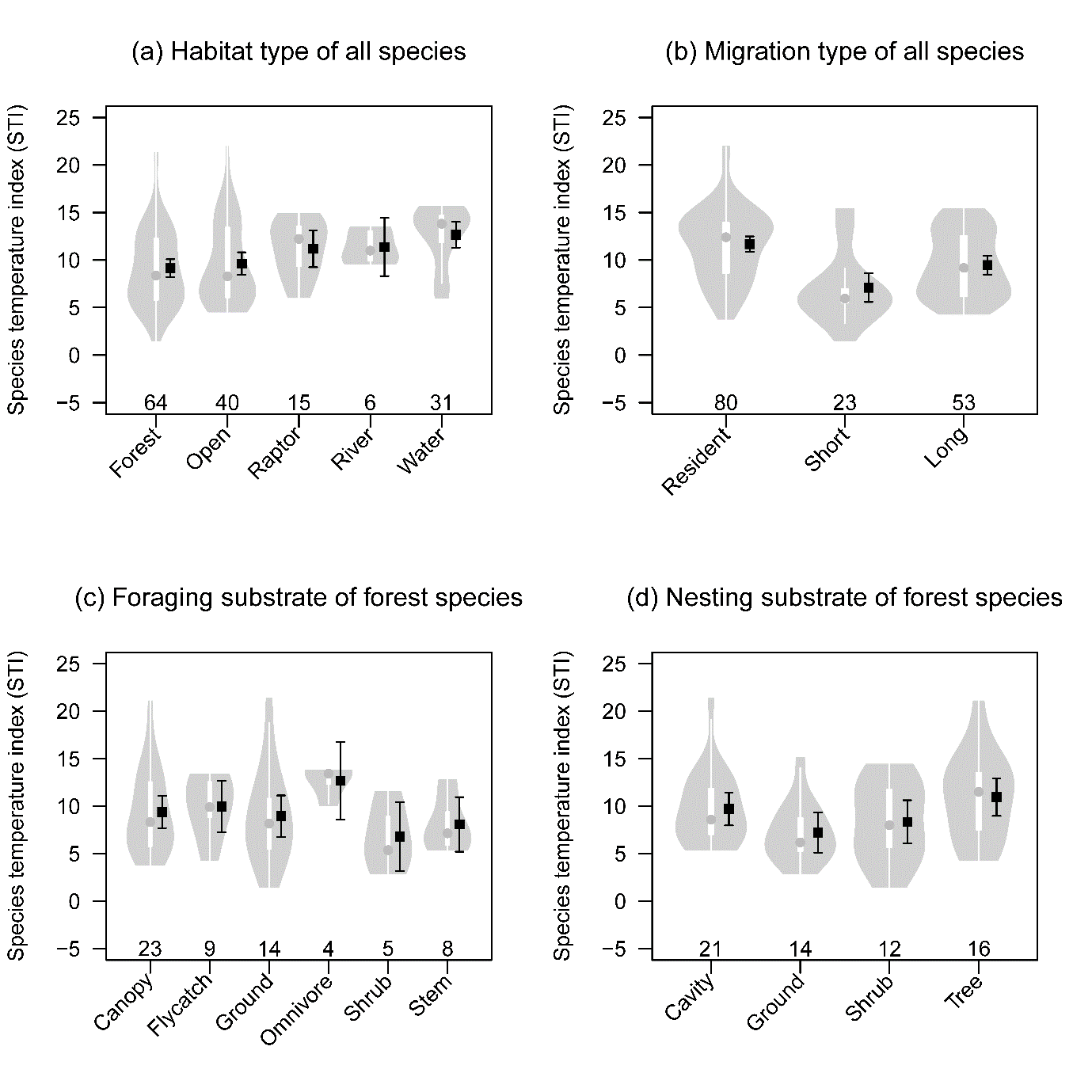


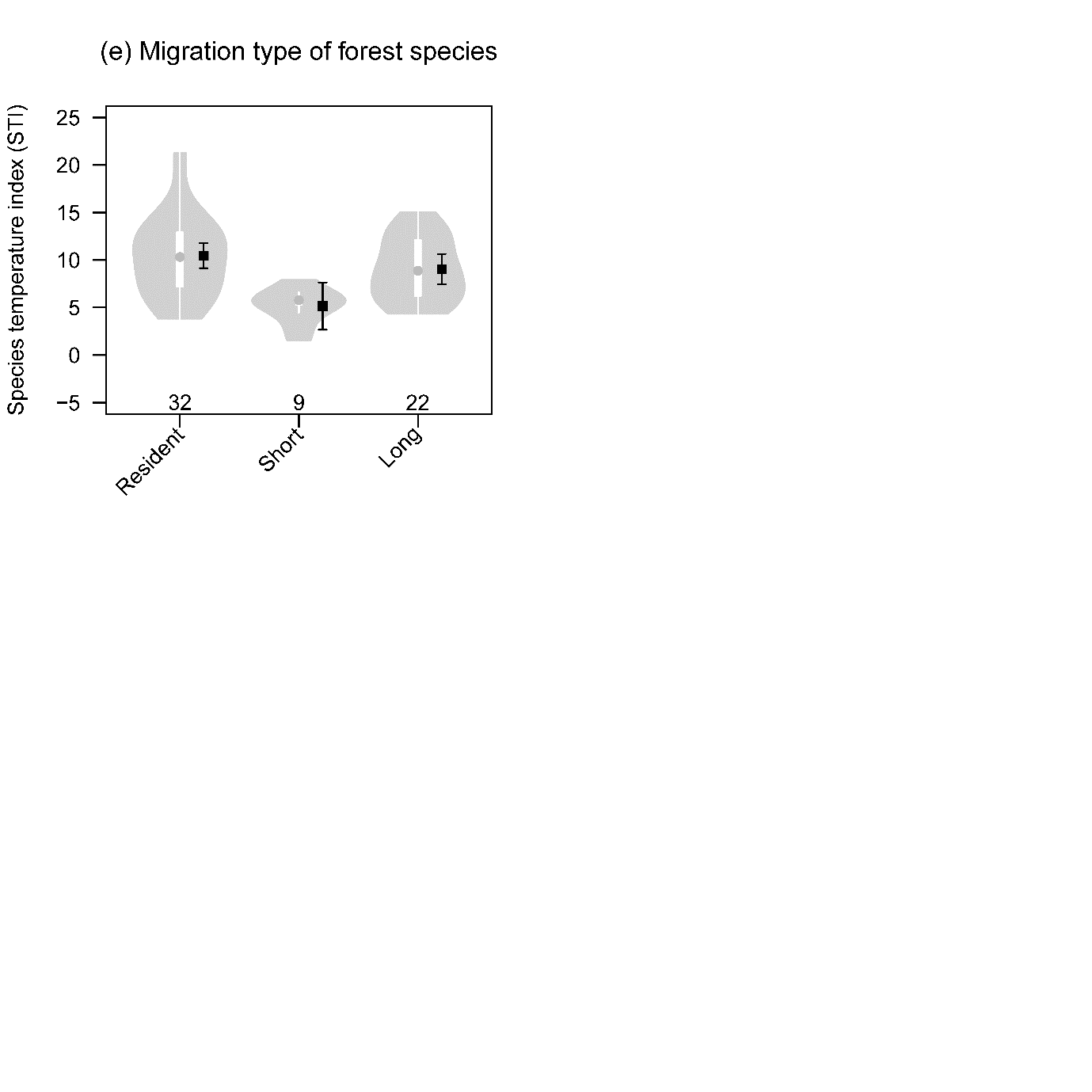


Fig. S2-1. Effects of ecological traits on STI, including (a) habitat types, (b) migration types, (c) forest foraging types, (d) forest nesting substrates, and (e) forest species migration types.

Distribution of the data are shown by violin plots indicating interquartiles, median, lower and upper whiskers. Mean estimates of individual groups and their 95% CIs are shown next to the violin plots and were obtained based on the OLS model by omitting the intercept in the “lm” function in R (cell-means model).


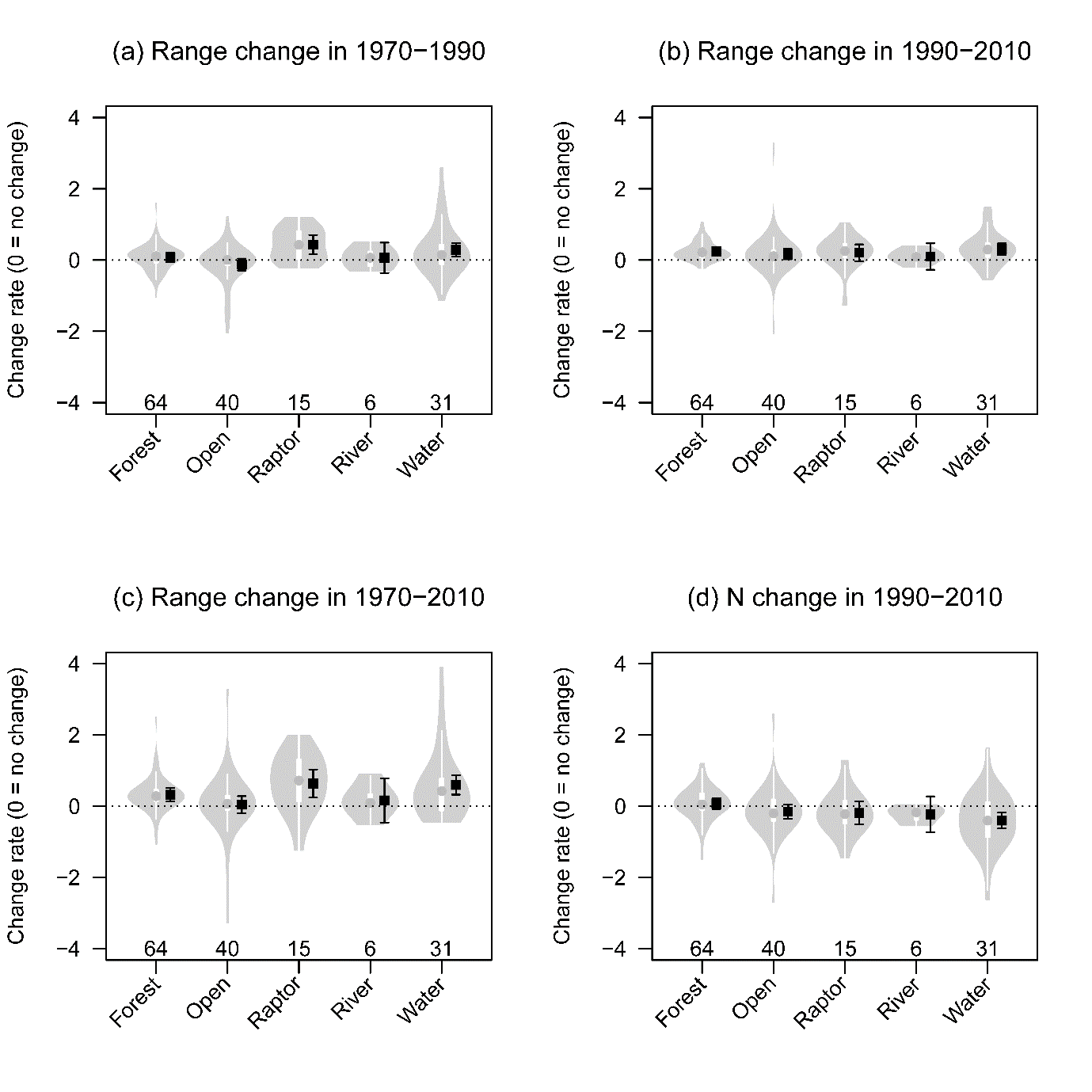


Fig. S2-2. Changes in (a, b, c) range size and (d) abundance for four habitat types from the (a) 1970s to 1990s, (b, d) 1990s to 2010s, and (c) 1970s to 2010s.

See Fig. S2-1 for details.


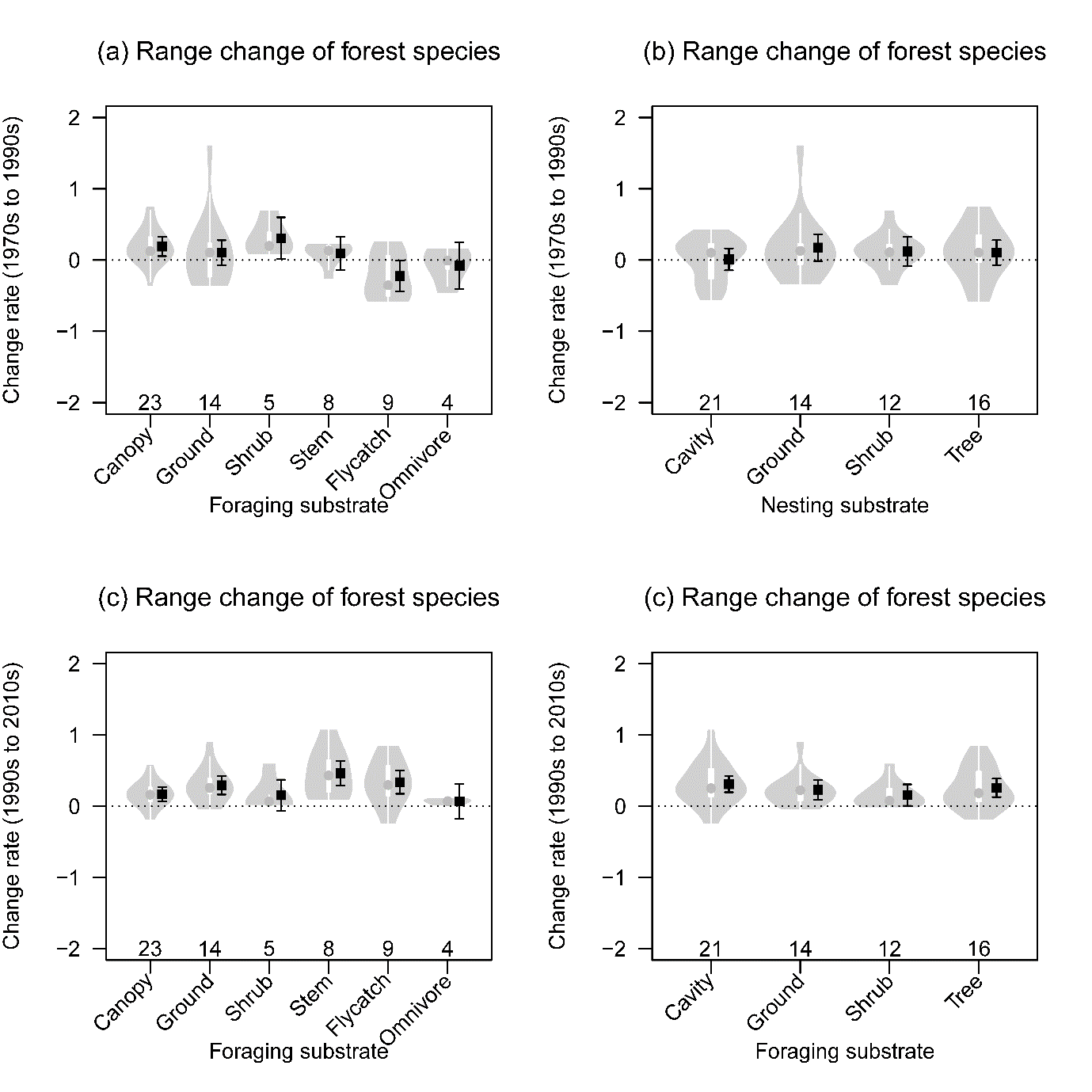


Fig. S2-3. Range changes from (a, b) the 1970s to 1990s and (c, d) the 1990s to 2010s for forest species in relation to (a, c) foraging types and (b, d) nesting substrates.


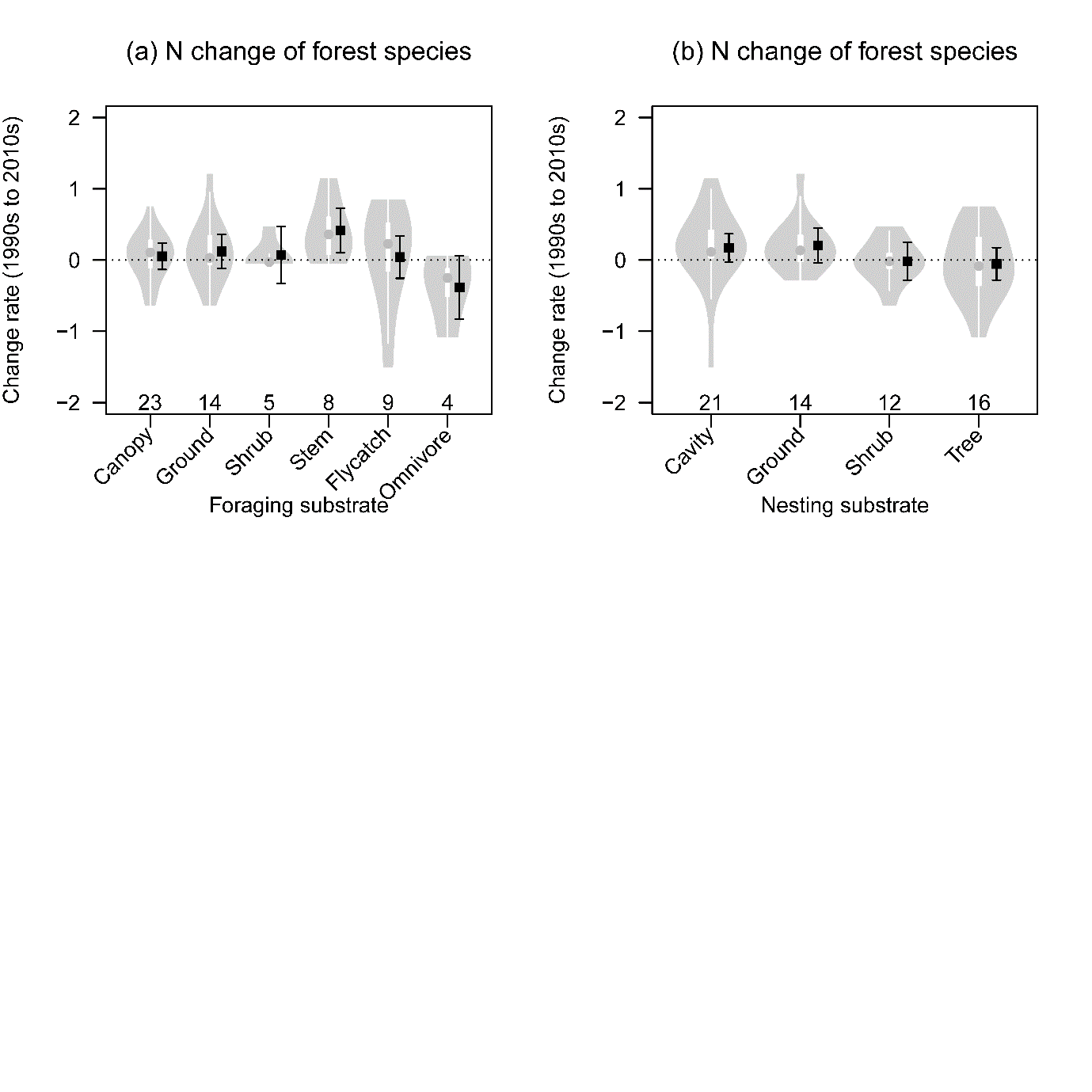


Fig. S2-4. Changes in forest bird species abundance from the 1990s to 2010s according to (a) foraging types and (b) nesting substrates.


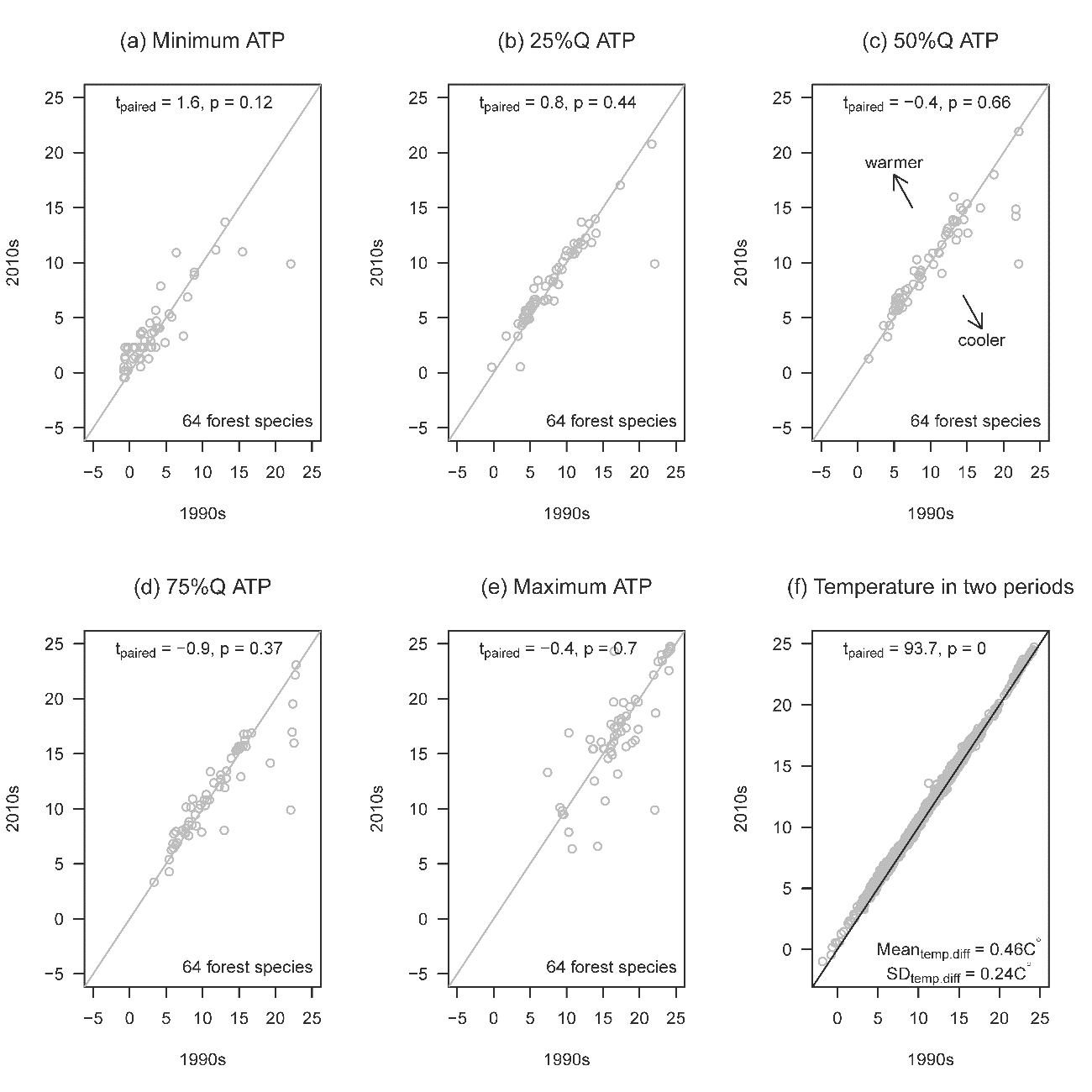


Fig. S2-5. Relationships between forest species distribution changes from the 1990s to the 2010s and the (a) minimum, (b) first quartile, (c) median, (d) third quartile, and (e) maximum survey-year annual temperature (ATP). (f) The survey-year mean annual temperature generally became warmer during the past 20 years.


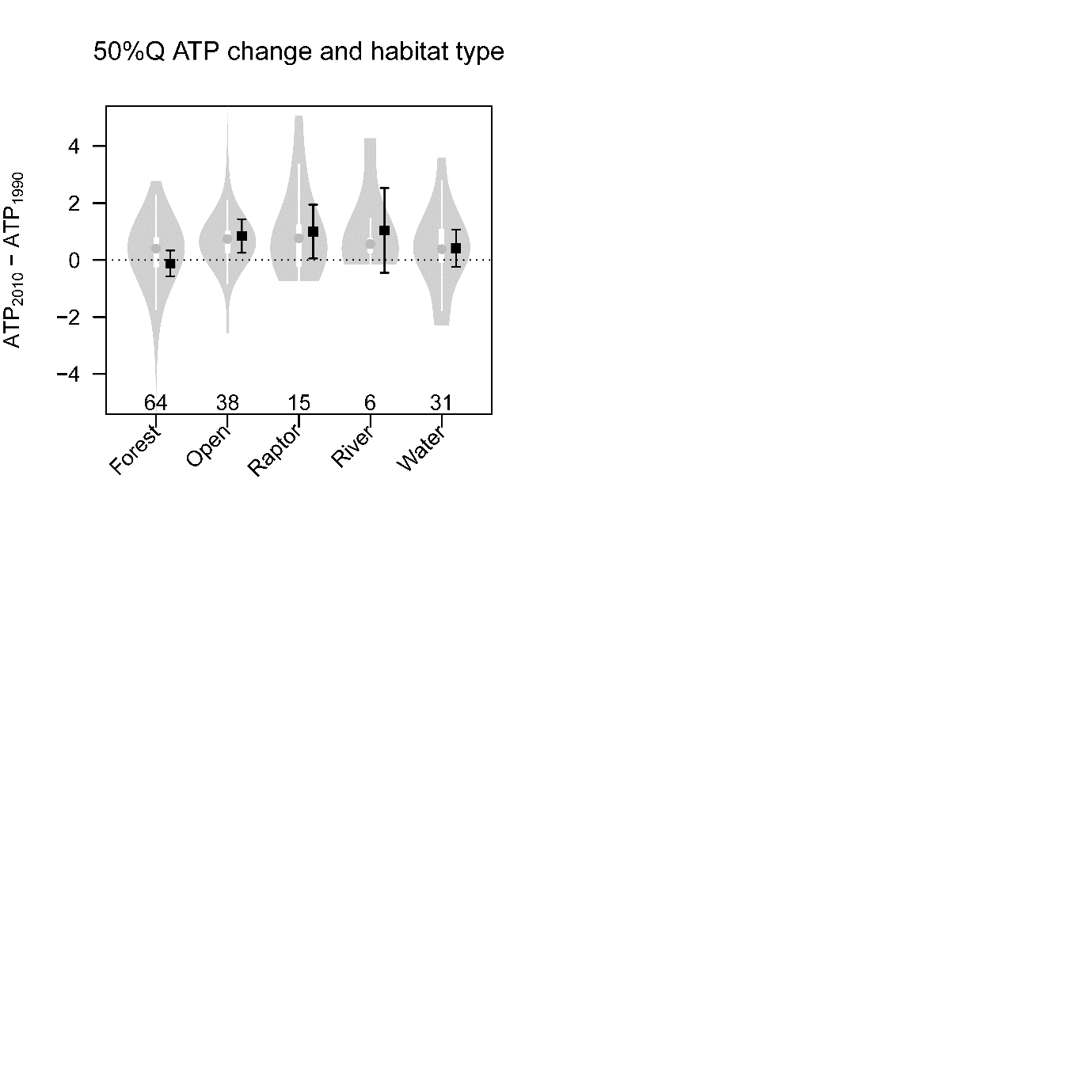


Fig. S2-6. Habitat type and distribution shifts in relation to survey-year annual mean temperature.

See Fig. S2-1 for details.


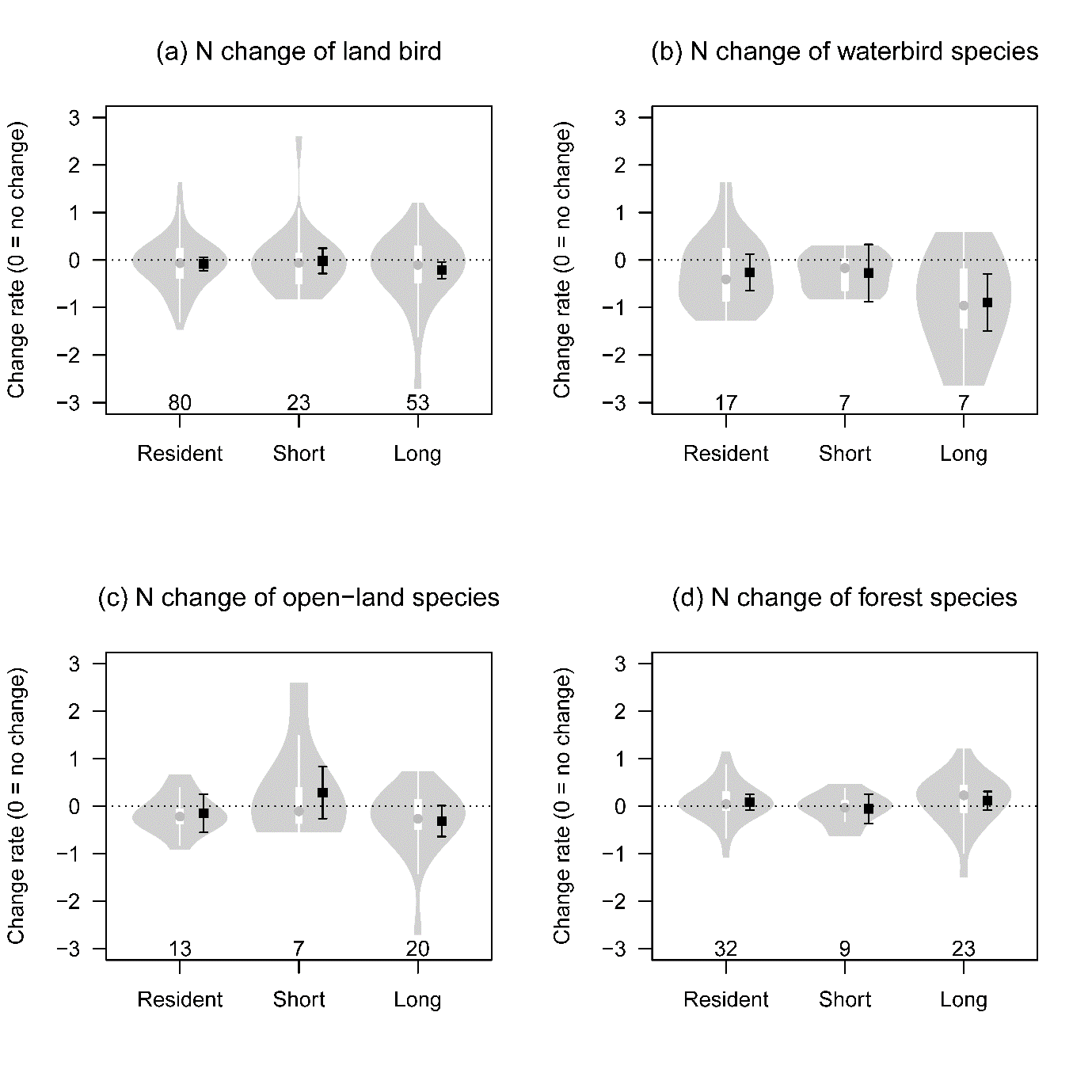


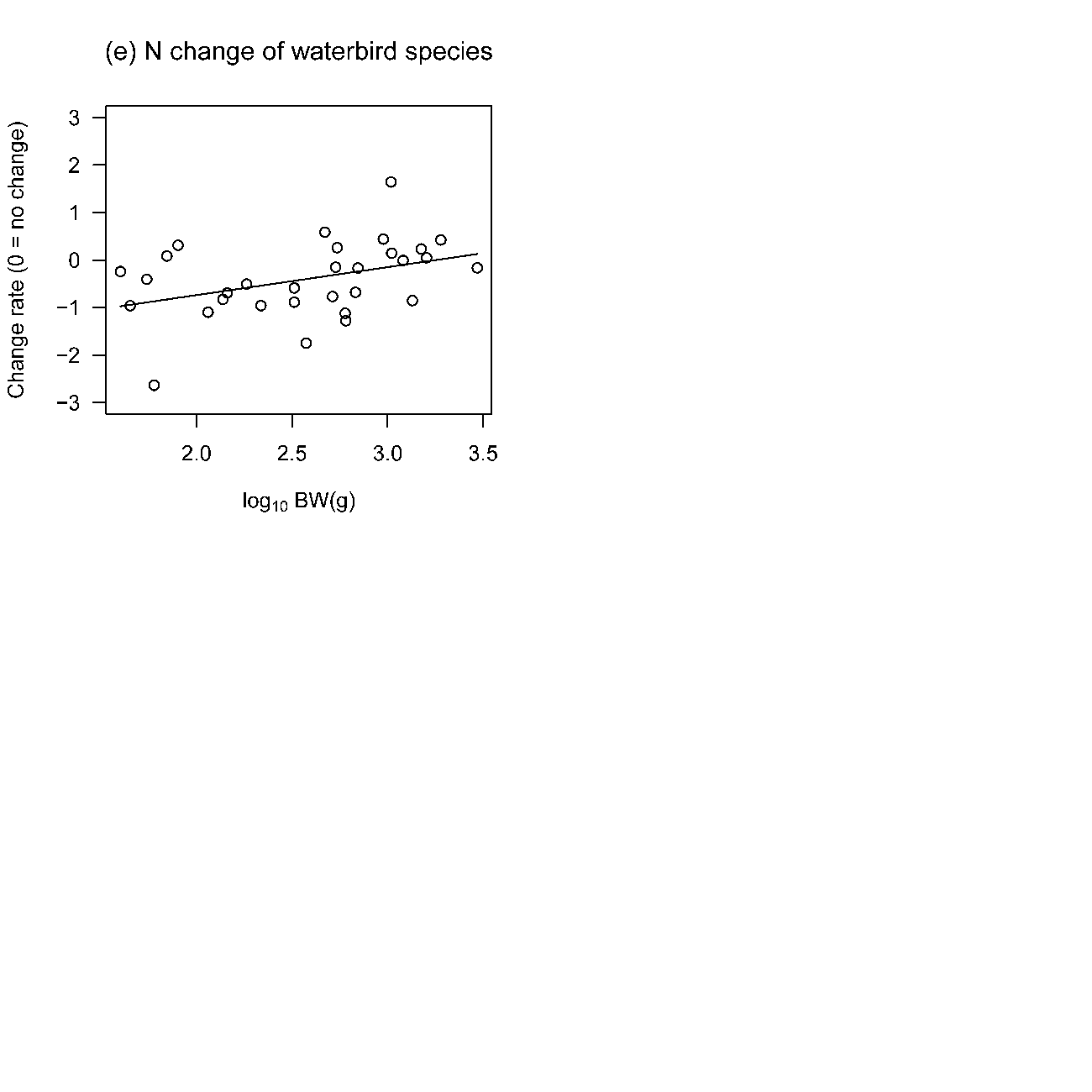


Fig. S2-7. (a–d) Changes in abundance in relation to migration type for (a) land, (b) waterbird, (c) open-land, and (d) forest species. (e) The best model explaining abundance changes among waterbird species included body weight as the sole covariate (see Table S2-2). Line indicates the line of best fit; the coefficient was significant (estimate = 0.59, standard error = 0.26, *t* = 2.28, *p* = 0.03).


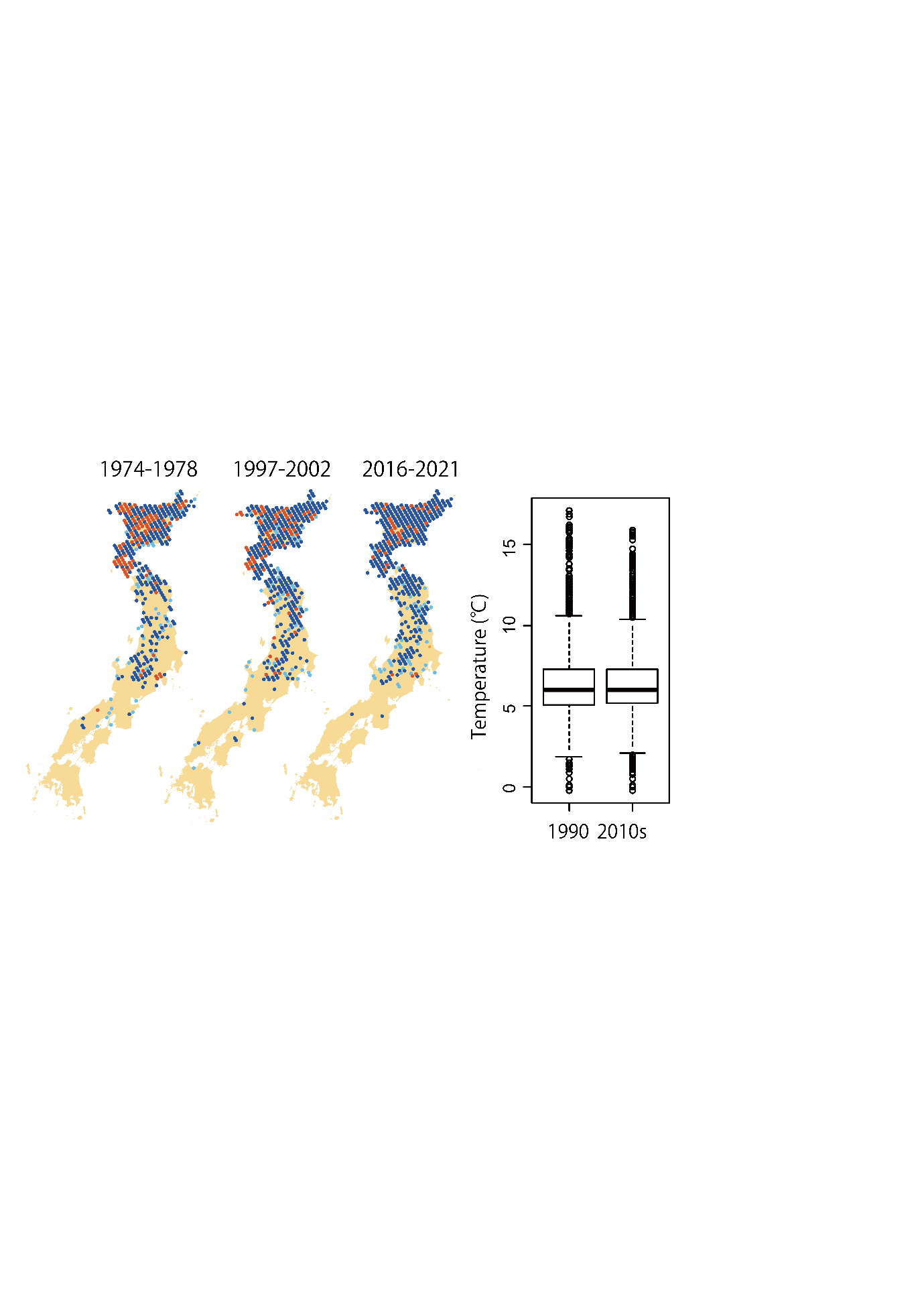


Fig. S2-8. Spatial distributions of black-faced bunting *Emberiza spodocephala* in the three surveys.

In the spatial maps, grids with occurrence data are marked by red, blue, and light blue indicating the reproductive ranks, namely, confirmed breeding, probable breeding, and uncertain breeding, respectively. This species is a common breeding bird species in northern Japan, and the latest survey (2016-2021) showed that their detection in warm areas (lower part of these figures) became rarer. This pattern is manifested in the disappearance of high values of long-term annual temperature within distributions (shown in the boxplots).


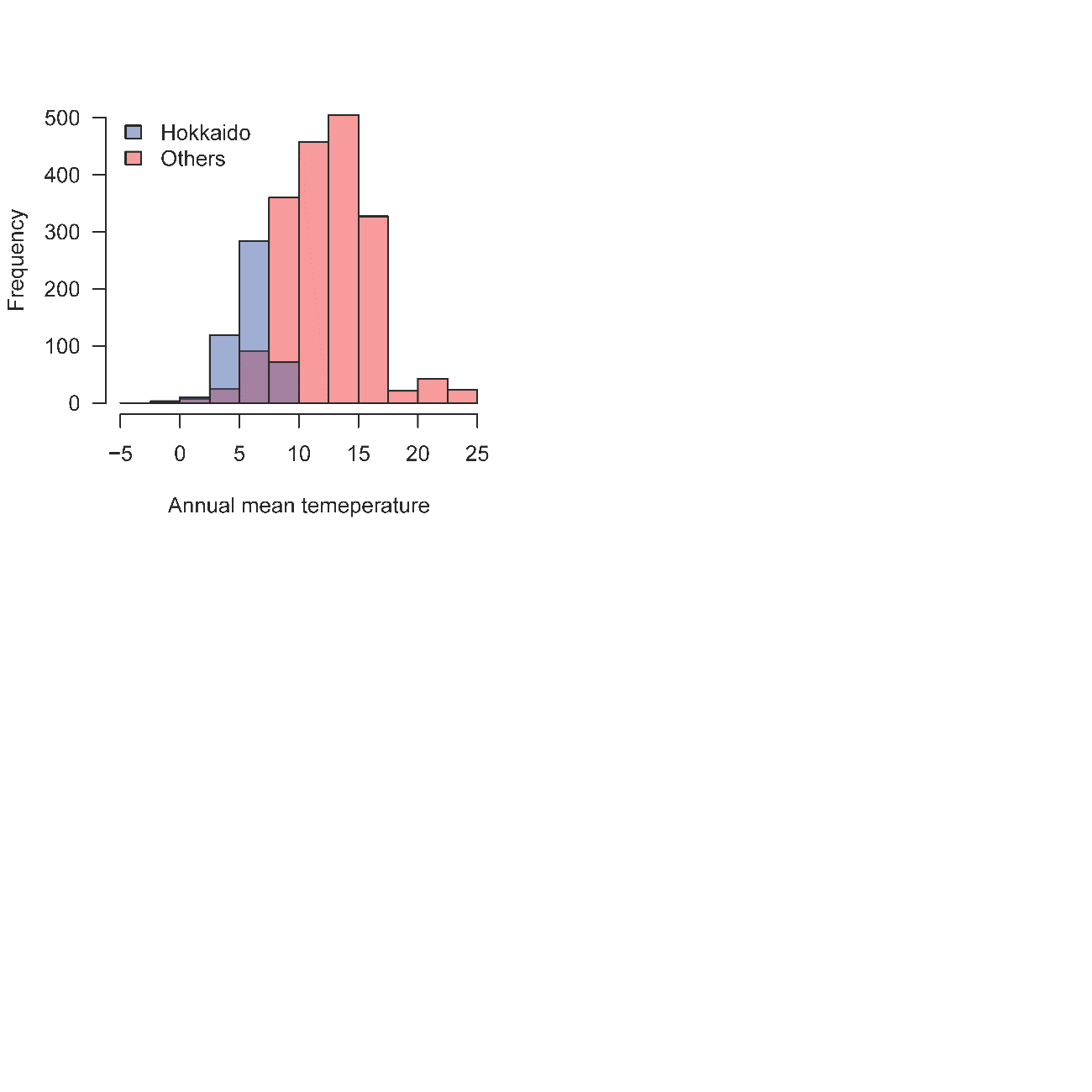


Fig. S2-9. Histogram of annual mean temperature (°C) averaged over 30 years for 2,347 transects in Japan. Because the transects were established systematically throughout the country, the temperature distribution represents available temperature niches in Japan.
